# A comprehensive review of microbial contamination in the indoor environment: sources, sampling, health risks, and mitigation strategies

**DOI:** 10.3389/fpubh.2023.1285393

**Published:** 2023-11-23

**Authors:** Hitikk Chawla, Purnima Anand, Kritika Garg, Neeru Bhagat, Shivani G. Varmani, Tanu Bansal, Andrew J. McBain, Ruchi Gulati Marwah

**Affiliations:** ^1^Institute for Cell Biology and Neuroscience, Goethe University Frankfurt, Frankfurt, Germany; ^2^Department of Microbiology, Bhaskaracharya College of Applied Sciences, University of Delhi, New Delhi, India; ^3^Department of Biosciences and Bioengineering, Indian Institute of Technology Roorkee, Roorkee, India; ^4^Department of Biomedical Science, Bhaskaracharya College of Applied Sciences, University of Delhi, New Delhi, India; ^5^Department of Biochemistry, All India Institute of Medical Sciences, New Delhi, India; ^6^School of Health Sciences, Faculty of Biology, Medicine and Health, The University of Manchester, Manchester, United Kingdom

**Keywords:** indoor environments, indoor air quality, public health, bioaerosols, microbial diversity

## Abstract

The quality of the indoor environment significantly impacts human health and productivity, especially given the amount of time individuals spend indoors globally. While chemical pollutants have been a focus of indoor air quality research, microbial contaminants also have a significant bearing on indoor air quality. This review provides a comprehensive overview of microbial contamination in built environments, covering sources, sampling strategies, and analysis methods. Microbial contamination has various origins, including human occupants, pets, and the outdoor environment. Sampling strategies for indoor microbial contamination include air, surface, and dust sampling, and various analysis methods are used to assess microbial diversity and complexity in indoor environments. The review also discusses the health risks associated with microbial contaminants, including bacteria, fungi, and viruses, and their products in indoor air, highlighting the need for evidence-based studies that can relate to specific health conditions. The importance of indoor air quality is emphasized from the perspective of the COVID-19 pandemic. A section of the review highlights the knowledge gap related to microbiological burden in indoor environments in developing countries, using India as a representative example. Finally, potential mitigation strategies to improve microbiological indoor air quality are briefly reviewed.

## 1 Introduction

Around 90% of our time is spent indoors due to lifestyle changes and work habits; thus, indoor air quality is closely related to our health and comfort (1, 2). Many reports confirm that the concentration of pollutants can be 2 to 5 times higher inside than outside (3). In addition to the chemical pollutants, biological contaminants, such as bacteria, viruses, fungi, insects, mites, pollen, and pet dander, are also present in the indoor environment (4, 5). Microbial contaminants in the indoor environment can exist on surfaces, as suspended cells, or as bioaerosols (6). They show variations in numbers and types depending on the kind of indoor environment, sources of contamination, and other environmental factors. These chemical and biological pollutants may adversely impact indoor air quality leading to Indoor Air Pollution (IAP).

IAP is considered one of the five top risks influencing public health and presents a much higher risk than outdoor pollution. An estimated 3.2 million people die every year due to IAP (7, 8). Poor indoor air can have both immediate and long-term health effects, which are commonly referred to as Sick building syndrome (SBS), Building-related illnesses (BRIs), and Multiple chemical sensitivities (1, 9, 10). SBS refers to a collection of symptoms reported by the occupants or workers of a given building and is generally not attributed to a particular cause (10). These may include immediate or short-term effects such as irritation of the eyes, nose, and throat, headaches, dizziness, and fatigue (11, 12). This may also affect the nervous and cardiovascular systems and cause reduced fertility and congenital disabilities in the long term (8). BRI’s on the other hand refers to medical conditions such as hypersensitivities, asthma, and respiratory infections such as pneumonia, linked to a specific cause (13). Generally, exposure to microbes or their components is associated with three main groups of illness: toxicity, infections, and allergic reactions, including respiratory infections and other related diseases (1, 14, 15). Neonates, young children, older adults, and especially people suffering from co-morbid conditions are highly vulnerable to IAP (2, 16).

Particularly in developing nations, including India, the situation is more critical because of overpopulation and other socio-economic factors (17, 18). Tackling indoor air pollution is challenging and requires interdisciplinary efforts (19). There remains a significant knowledge gap and a lack of standards and guidelines, especially for assessing the microbiological quality of indoor air (20). Since the COVID-19 pandemic, IAP has been brought to the forefront because, globally, people are working remotely and spending more time indoors (21).

**Figure 1:**
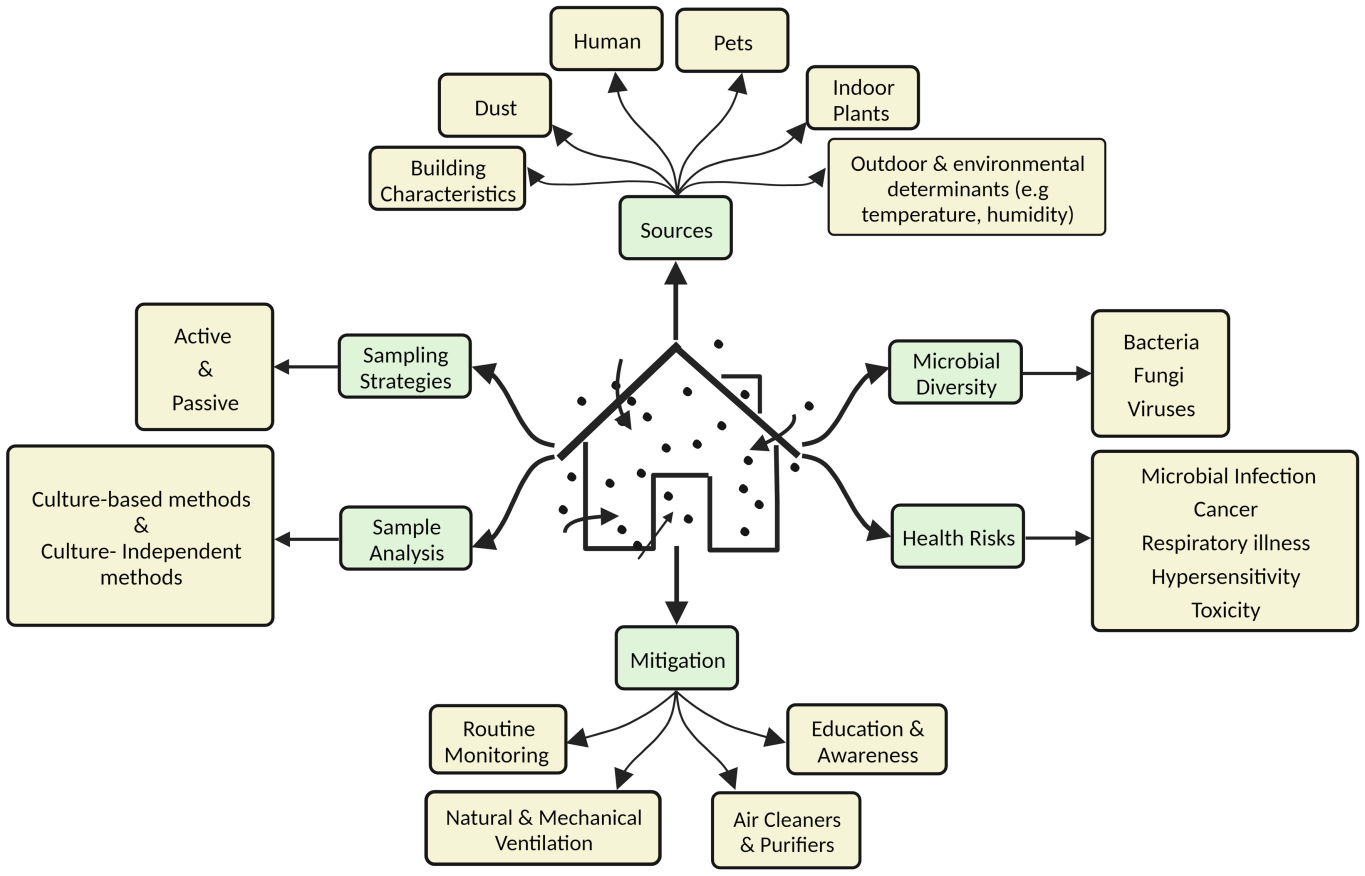
Various aspects of microbiological contamination of indoor air.

This review provides a comprehensive overview of microbial contaminants in diverse indoor environments, their sources, sampling and assessment strategies, the factors affecting their prevalence, and the associated health risks (Figure 1). The significance of IAP in the context of the COVID-19 pandemic is also highlighted. The severity of the problem is highlighted for developing countries with a particular reference to the scenario in India. The last section discusses various strategies to mitigate this challenge.

## 2 Methodology to conduct literature search

A thorough search of academic databases such as Google scholar and PubMed was conducted. We searched for specific terms such as ‘Microbial Indoor air quality,’ ‘Indoor air and Bioaerosols,’ ‘Bacteria in indoor air, ‘Fungi and molds in Indoor air,’ ‘Microbes in Indoor air and health’. The titles and abstracts of the articles were screened and assessed for the inclusion in the review. Articles were cross checked, duplicated and irrelevant articles were excluded. Further, relevant papers from the reference list of the included papers were also considered to ensure maximum coverage of the literature. A list of 430 papers were extensively discussed among the authors to resolve any discrepancies and reach a consensus. Finally, 314 references were included in this review, out of which 76% are from 2010 onwards.

## 3 Sources of microbial contaminants in indoor air

Outdoor pollutants influence the number and kind of microorganisms that enter the indoor environment displaying a source-sink relationship (22, 23). Apart from these, various inherent sources in the built environment contribute to the abundant microbial numbers and variety. Abiotic factors like moisture, relative humidity, and temperature can influence indoor microbiology (24, 25).

### 3.1 Humans

Human occupancy and their activities affect the microbial numbers and diversity in the indoor environment (25, 26). In the indoor air, respirable particulate matter and bacterial DNA increased with human occupancy (27). It has been estimated that humans shed approximately a billion skin cells daily, likely influencing microbial concentrations in the indoor air (28). Abundant bacteria and viruses from human oral and respiratory fluids can become aerosolized via talking, breathing, coughing, sneezing, etc. and reach varying distances depending on the droplet size (29). The frequency of cooking, vacuum cleaning, showering, and other human activities also affect microbial numbers and diversity (30). Patients, doctors, visitors, and hospital staff and their activities contribute to the pathogen load in the hospital environment (5, 31). Fungal concentrations have been reported to be elevated due to increased human movement from outside to inside, specifically linked to increased dust levels (32).

### 3.2 Pets

Pets also influence the indoor air microbiome. Animal skin, saliva, hair, fecal matter, and fleas are expected to contribute to microbial diversity in indoor air (28, 33, 34). Bacterial diversity and community richness have been reported to increase in households owning dogs and cats (35, 36). In a study involving 70 pet dogs, about 44 fungal isolates were obtained from the hair and skin of these animals (37).

### 3.3 Dust

House dust consists of hair, cotton fibers, bacteria, molds, and other particulate matter (38–40). Dust-borne microbes can become resuspended into indoor air, increasing the risk of inhalation (41). Dust is reported to be dominated by skin-associated Gram-positive bacteria (35, 42, 43). Leppänen et al. (44) quantitatively assessed 259 house dust samples from both rural and urban homes in Finland. This study indicated that the fungal composition and seasonal variations correlated well between indoor air, settled, and reservoir dust samples. The latter showed reproducibility in repeated sampling over time. In another study by Wu et al. (45), bacterial composition and diversity in indoor dust samples were found to be affected by abiotic factors in the indoor environment. Marked differences in bacteria isolated from dust samples collected from university dormitory rooms and printing shops were reported. Human activities or strong air currents can also suspend respiratory viruses deposited in dust or indoor surfaces (46).

### 3.4 Building characteristics

Building design and maintenance, moisture build-up, and inadequate ventilation are common triggers for microbial build-up in the indoor environment (47). Plumbing systems can impact indoor air quality by adding bioaerosols to the built environment. Toilet flushing can generate large numbers of aerosolized bacteria from human faces, especially in case of poor ventilation, and if pathogenic, they carry the risk of transmission to healthy occupants (48, 49). In a toilet-seeding experiment by Barker and Jones (50), colony-forming units of *Serratia marcescens* rose sharply from 0 to 1,370 CFU/m^3^ after the first flush. Plumbing faults or leaks, water-damped carpets, ceilings, walls, and cramped building design can lead to increased dampness in the indoor environment, further increasing mold growth, odor, and microbial counts (47, 51). Additionally, the events of flooding can substantially increase the dampness inside houses which may favor the growth and dispersal of mold and bacteria. This was observed in the aftermath of flooded houses due to hurricane Katrina in New Orleans, Louisiana, United States (52). Construction and other materials used in buildings significantly contribute as potential sources as they can degrade and convert into organic compounds that support the growth of microorganisms (5). Both natural and mechanical ventilation practices influence the microbial ecology of indoor air (26, 53). Heating, ventilation, and air-conditioning systems (HVAC), primarily installed for air exchange, cleaning, and thermal comfort, can favor microorganisms’ growth if not properly maintained and cleaned (54). These microbes can remain viable on the internal filters of air-conditioning units for a long time. They can re-enter the indoor environment due to filter clogging, inefficient operation, improper maintenance, and malfunction (55). Further, leakage or condensation can wet the filters, favoring mold and bacterial proliferation (56).

### 3.5 Indoor plants

Microbial diversity and numbers are significantly contributed by indoor plants in the built environment (57, 58). Indoor plants can increase humidity levels and favor the excessive growth of molds and other airborne microorganisms (59). Due to agitation from the watering of plants or through the generation of strong air currents from fans, levels of airborne fungi were found to increase (60).

## 4 Sampling strategies

There is a lack of international consensus and standard operating procedures for collecting samples from indoor environments for both qualitative and quantitative estimation of microbial contaminants. International standards, including ISO 16000 series such as 16,000-17, 16,000-18, 16,000-19, 16,000-34, offer guidelines concerning sampling techniques, kinds of samplers, and sample analysis (47, 61, 62). Indoor air and dust samples are mainly used for microbial assessment of the indoor environment. Dust samples could be settled dust (on various surfaces like floors and tables) or reservoir dust (in mattresses, carpets, and bedding) (44). Dust collection is a quick but indirect method for sampling bioaerosols in the indoor environment. Various kinds of passive and active sampling techniques are widely and routinely used for the assessment of microbial contamination of indoor air.

### 4.1 Passive sampling

Passive sampling using ‘agar settle plates’ is one of the most widely practiced procedures to collect settled dust samples under the force of gravity. The air sample is collected according to the 1/1/1 scheme, plates are incubated, and results are expressed as CFU/m^3^ using the equation described by Omeliansky (63, 64). It is a simple, inexpensive, and unobtrusive sampling method (65). It gives comparable results, requires no special powered instruments or personnel, and is not influenced by engineering factors. Further, it provides a valid risk assessment if passive sampling is performed in an operation theater or near a surgical site (63, 66).

A simple and cost-effective dustfall collector developed by Wurtz et al. (67) is useful for prolonged airborne dust collection to measure significant concentrations of culturable fungi. Passive samplers based on electrostatic attractions, such as electrostatic dust clothes and the recently invented Rutgers electrostatic sampler, are also used (68, 69). Also, a vacuum cleaner can collect reservoir dust like those from mattresses/carpets or settled dust samples above floor surfaces (44, 70). Swabs are used to collect surface dust from table tops, computers, doors, walls, and cupboards. However, it allows only a qualitative measure of microorganisms’ airborne concentrations, and the sample’s age is unknown (47).

### 4.2 Active sampling

Active sampling collects airborne microorganisms present in inhalable dust in the indoor environment. In this, an air sampler physically draws a pre-set volume of air with the help of a pump, through or over a particle collection device into a liquid or solid culture medium or a nitrocellulose membrane (71). Various types of active air samplers like Anderson, Active Casella slit, Surface air system, and Coriolis cyclone samplers are available commercially based on sampling techniques such as filtration, impaction, impingement, and cyclone, each with its own sets of advantages and limitations (4, 61, 65). Active samplers require trained operators and a power supply, which might constrain their use in remote areas. Frequent replacement of collection media can help avoid reducing sample viability and overloading (65). Massoudinejad et al. (72) stated that active sampling was more sensitive and precise than passive sampling. This sampling technique is mainly applicable when the concentration of microorganisms is not very high, such as in an operating theater of a hospital (73).

These two sampling procedures have shown a good correlation in some studies (68, 74, 75) while others reported no correlation (70, 73, 76, 77). These variations in results could occur due to the type of sample and sampler used, the volume of air sampled, and the place or time of sampling (61, 78). Frankel et al. (79) pointed towards an urgent need for standardized sampling methodologies as they observed significant differences in the levels of culturable fungi, bacteria, endotoxin, and total inflammatory potential, in various air and dust samples collected from homes.

## 5 Sample analysis

**Figure 2:**
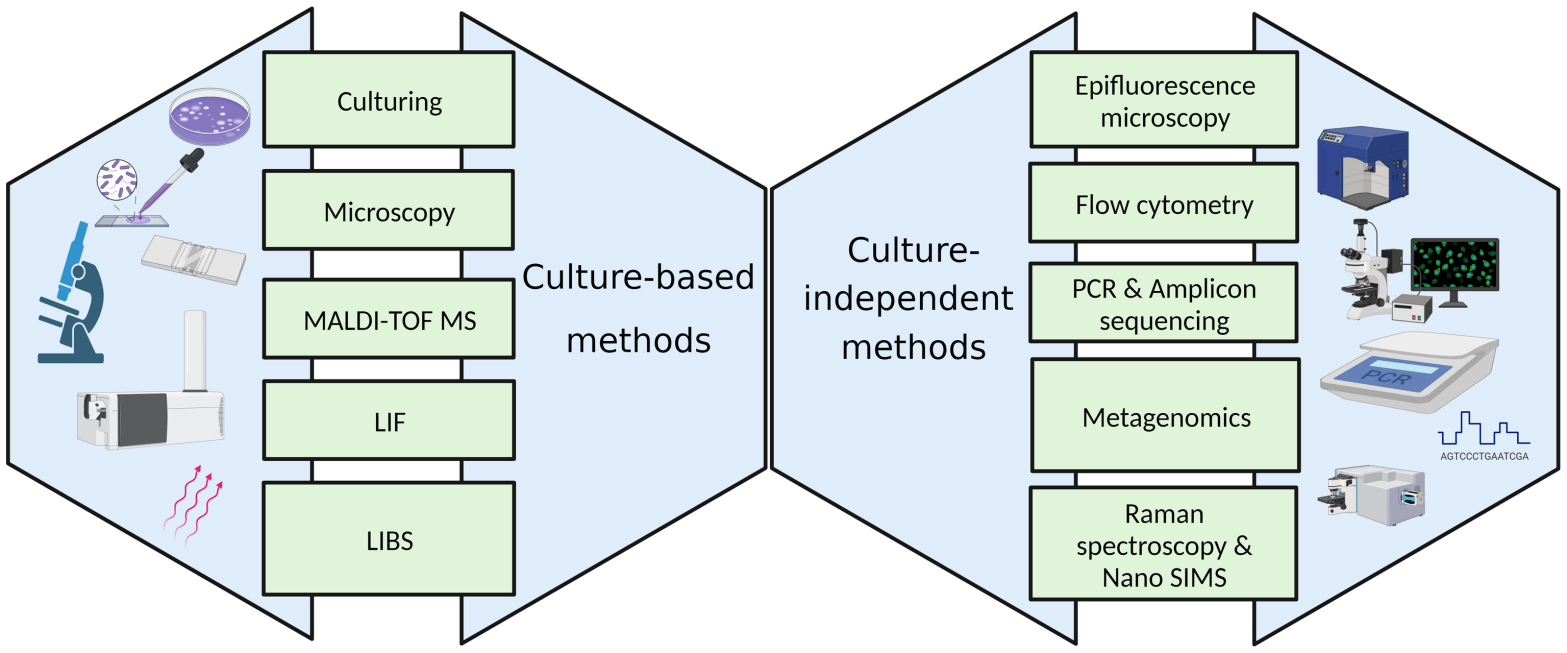
Techniques for the microbiological analysis of the indoor environment.

Various culture-based and culture-independent methods employed to enumerate and identify microorganisms in the indoor environment are represented in Figure 2.

### 5.1 Culture-based methods

#### 5.1.1 Culturing and microscopy

Microbial culturing techniques are simple, traditional, low-cost, and well-developed, primarily involving different types of microbiological media. Bacteria are routinely grown on general-purpose media such as nutrient agar, tryptic soy agar, blood agar, and casein soy peptone agar (80, 81). In addition to this, various selective media such as endo agar, eosin methylene blue agar, and mannitol salt agar are also employed (82). This method provides qualitative and quantitative measures of culturable bacteria and population diversity. Several broad-spectrum complex media, such as potato dextrose agar, malt extract agar, rose bengal agar, and dichloran glycerol 18 agar are used for fungal isolation and quantification (80, 81). Compared to bacteria, enumerating fungi is more complex and thus limits the comparison of data from different studies (7).

After processing the sample and using appropriate staining techniques, identification and enumeration of bacterial or fungal contaminants can be carried out by bright field microscopy (6). Alternatively, airborne microbial particles can be directly sampled onto glass slides, semisolid media, or membrane filters and microscopically examined (4). For enumeration and identification of fungi and their spores, hemocytometer chambers and adhesive tapes appressed onto indoor surfaces are also combined with microscopy (83). Both culturing and microscopic techniques are however time-consuming and tedious. Culturing methods show intrinsic constraints in obtaining actual microbial numbers and diversity. Direct microscopic counts are generally exceedingly higher than viable colony counts, commonly phrased as ‘the great plate count anomaly’ (84).

#### 5.1.2 Matrix-assisted laser desorption/ionization time-of-flight mass spectrometry

MALDI-TOF MS is a highly reliable and rapid spectrometric technique compared to traditional microbiological and molecular methods and is thus useful for high-throughput microbial identification (85). In this, the mass-to-charge (m/z) ratio of ribosomal protein/peptide (analyte of interest) of the given microorganism is measured by determining the time required for it to travel the length of the flight tube following ionization with a laser beam (86). A unique mass spectrum called peptide mass fingerprint is obtained for the organism quickly. Identification is made using an algorithm-based approach from databases containing the MS reference spectra of peptides and proteins extracted from known microorganisms (87, 88). This technique is gaining popularity for identifying bacteria and fungi from indoor dust and air samples (89–91). The microbial culture is transferred directly onto the sample target from the culture medium. MALDI-TOF MS is simple, cost-effective, and does not require highly skilled personnel (92).

#### 5.1.3 Laser-induced fluorescence and laser-induced breakdown spectroscopy

LIF spectroscopy is a relatively novel technique for non-contact real-time online detection of bioaerosols. In this technique, the given bacterial or fungal culture is excited by laser light, showing the largest cross section with the selected wavelength (4, 93). Following de-excitation, the light of a longer wavelength is emitted, which is recorded by a photomultiplier tube resulting in the generation of a spectral fingerprint for each microbial species. Bioaerosols can be differentiated from other airborne particles based on their distinct fluorescence spectra and assessed quantitatively based on fluorescence intensity (94). It efficiently detects microbial contamination of surfaces in industrial and hospital cleanroom facilities, the food-processing industry, operating theaters, and as a biosafety measure in microbiological or medical laboratories or cases of suspicion of a deliberate or accidental release of biological warfare agents (95, 96). However, not all excited species may fluoresce, causing improper measurements (4). This technique has also been recently investigated for the first time for detecting Picorna viruses, thus showing the potential for rapid virological analysis with a substantial cost reduction (97, 98).

LIBS is yet another rapid, flexible, and real-time monitoring technique based on the unique atomic composition of plasma produced when a high-power pulsed laser is focused on a minimal area of the sample surface (99). The sample gets ablated, generating a plasma plume with temperatures over 100,000 K, breaking the sample into excited ionic and atomic species. The plasma then expands and cools within a short time, emitting radiation. Thus, the characteristic emission lines give information about the sample (4). Saari et al. (94, 100) demonstrated the potential of LIBS and LIF techniques as promising tools for the online detection and differentiation of bioaerosol types.

### 5.2 Culture-independent methods

#### 5.2.1 Epifluorescence microscopy

Samples of airborne microbes collected in liquid buffer solutions or on membrane filters from the indoor environment are stained with fluorescent dyes and counted using epifluorescence microscopy (81, 101). DAPI and Acridine orange are popularly used for bioaerosol monitoring (4). It is a high throughput automated technique that allows the counting and differentiation of viable cells of culturable and non-culturable bacteria and fungi from non-viable cells or particles. Compared to conventional culturing, this technique gives much higher estimates of airborne microbial numbers for bacteria and fungi (102, 103), which was confirmed by Chi and Li (104) in a study conducted on bioaerosol concentrations and viability in swine buildings. However, the technique has the limited ability to differentiate between bacteria and fungi in case of size overlap and gives false positive results if the dye binds to the organic material (4, 102).

#### 5.2.2 Flow cytometry

Flow cytometry is a rapid real-time technique for enumerating, detecting, and sorting microscopic particles suspended in a fluid stream with a high sample throughput (105, 106). It has been widely used for microbial analysis of environmental samples (107, 108). Different cell types cannot be distinguished only based on light scattering characteristics for bioaerosols, so using fluorescent dyes are recommended with this technique (106). After collecting indoor air samples in a liquid, flow cytometry with fluorescence is applied to quantify cells (4). The sample is hydrodynamically focused to create a single cell stream that passes in front of a laser beam, and the fluorescence emitted by each particle is then measured with photon detectors (105). The combination of FCM with fluorescence has been used to assess bacterial counts in hospital wards, reporting significantly higher counts than culture-based methods (109). This method can also detect and quantify airborne fungi (83). The significant drawbacks of FCM are high cost, the need for highly skilled labor, low-temperature requirements, and false positive results due to abiotic particles (4).

#### 5.2.3 Single cell-based techniques: Raman microspectroscopy and nanoscale secondary ion mass spectrometry

Normal or spontaneous Raman spectroscopy and its variations have been used for the spectroscopic analysis of biochemical components of microorganisms. It is an effective, non-invasive, and label-free technique to extract the chemical fingerprint of an individual microbial cell (110). However, the spectra are obscured in standard Raman spectroscopy due to intrinsic fluorescence. Also, a burning effect is caused due to high-energy UV photons in UV resonance Raman enhancement spectroscopy (111). Surface-enhanced Raman spectroscopy (SERS) increases the Raman signal by suppressing the fluorescence and is effective under infrared and visible excitation and has thus emerged as an upcoming rapid, cultivation-independent technique for selective and real-time detection of single microbial aerosols (111, 112). The method involves adsorption or proximity of the analyte to a roughened gold or silver surface or nanoparticles. Following excitation by the incident light, the total enhancement of the SERS process is due to chemical and electromagnetic enhancement effects that generate a sizeable spectroscopic signal for the analyte (113). The major limitation is the reproducibility of the spectral data, as the laser has to strike at a point where the substrate and the analyte are present at an appropriate geometry. This limitation is overcome by combining Raman spectroscopy with other techniques. One study involved the combination of powerful magnification provided by scanning electron microscope in conjunction with the Raman interface to successfully target SERS active regions of the sample matrix to produce reproducible spectra (113).

Similarly in another study, bioaerosols were impacted and transferred to colloidal silver nanoparticles to obtain efficient Raman spectra (111). Schwarzmeier et al. (114) employed a Coriolis μ wet particle sampler with SERS to detect *E. coli* aerosols, facilitated using an advanced microarray readout system. Distinct Raman spectra of spores from several fungi associated with a damp indoor environment were obtained, suggesting the need to develop a library of Raman spectra for maximal differentiation of fungal spores at the species level (115).

NanoSIMS uses a sufficiently small beam size of approximately 50 nm to analyze single microbial cells or their parts. It combines features of microbial imaging techniques, stable isotope probing, and molecular biomarkers to study environmental microbial communities and draw comparisons (116, 117). Both Raman microspectroscopy and NanoSIMS, though have the potential to dissect and compare microbial communities in environmental samples but have not been used widely for profiling microbes in the indoor environment.

#### 5.2.4 Molecular methods

Several culture-independent molecular methods have vastly improved the quality of data obtained for indoor air microbiomes in terms of greater microbial abundance and diversity and thus have changed the way we think about built environments (118, 119). The polymerase chain reaction is the most widely used molecular technique for environmental microbial samples. It is frequently used to amplify genes coding for 16S rRNA for prokaryotes and 18S rRNA and internal transcribed spacer region (ITS) for eukaryotes (28, 120). This technique is rapid and particularly useful when concentrations of airborne microorganisms are low. Indoor airborne bacteria and fungi associated have been analyzed by conventional PCR in many studies (22, 53, 121). In recent years, quantitative PCR (qPCR), also called real-time PCR, has gained popularity in providing accurate data on total microbial numbers in environmental samples (122). It has been used to assess bacterial (123) and fungal concentrations and types (70) in indoor air and dust samples. The limitations of PCR include a prior requirement of sequence knowledge to design the primers and non-specific binding of the primer to similar sequences on template DNA (124).

Amplicons obtained from PCR can be further analyzed using various genetic fingerprinting techniques like amplified ribosomal DNA restriction analysis, terminal restriction fragment length polymorphism (T-RFLP), automated ribosomal intergenic spacer analysis, denaturing gradient gel electrophoresis, temperature gradient gel electrophoresis, single-strand conformation polymorphism, and denaturing high-performance liquid chromatography (125). These techniques are widely used for studying microbial communities in environmental samples and have also been applied for indoor air microbial profiling (116). For instance, in a study by Weikl et al. (126) involving T-RFLP, the variation, and diversity of bacterial and fungal microbiomes were studied in dust samples from 286 households. The study indicated that both bacterial and fungal communities followed a temporal and seasonal pattern, with indoor and outdoor determinants affecting the fungal microbiome more strongly.

Our knowledge of indoor air microbiome diversity and community dynamics has been substantially enhanced by metagenomic-based sequencing, having the advantage of rapidly analyzing millions of samples in parallel with high sensitivity (127). The classic first-generation Sanger sequencing is often replaced by advanced sequencing approaches using platforms such as the second-generation Illumina MiSeq/HiSeq and Ion Torrent systems, third generation PacBio SMRT sequencing, and recently commercialized fourth-generation Oxford Nanopore MinION technology (128, 129).

These advanced identification methods are being increasingly applied to analyze microbial diversity in indoor air and dust samples (130, 131). In a study, indoor air samples from elementary schools and day-care centers in Korea showed the presence of a wide variety of taxa in microbial communities previously not identified by culture-based methods (132). Nygaard et al. (128) characterized microbiomes in the building-dust by comparing Nanopore MinION and Illumina MiSeq 16S rRNA gene sequencing. This study did not find significant differences in microbial composition between the two methods; however, a greater taxonomic resolution was achieved with MinION technology. Diverse indoor airborne viral communities associated with disparate hosts were also observed using metagenomic sequencing of dust samples obtained from the HVAC filters by Rosario et al. (133).

However, metagenomic-based sequencing studies of the indoor environment are limited by the availability of reference sequences in genome databases, low biomass of microbial aerosols leading to poor DNA extraction, and missing out the DNA of microbes present in low-abundance. Also, a significant deterrent to adopting these advanced methods for routine microbial surveillance is offset by the high cost of resources and machinery, especially in low- and middle-income countries (134, 135).

## 6 Microbial diversity of indoor air

The indoor microflora is complex and dynamic, with their numbers and diversity guided by their sources and associated environmental factors. There are no uniform standards for the acceptable levels of microbial contaminants in the indoor environment. A study by the WHO expert group to assess the health risks of biological agents in indoor environments suggested that the total microbial concentration should not exceed 1,000 CFU/m^3^ (47). However, some recommend 300 CFU/m^3^ and 750 CFU/m^3^, the maximum limit for fungi and bacteria (136). Also, the standards followed may vary from one country to another and from one kind of indoor environment to another. Table 1 provides an insight into the vast diversity of microflora in various indoor environments such as homes, offices, schools, universities, libraries, healthcare facilities, shopping centers, public restrooms, and gyms. In comparison to bacteria and fungi, there are limited studies on viruses.

**Table 1:** An overview of the microbial diversity in different indoor environments (2017-2023).

S. No.	Type of Indoor Environment/Region	Source/Sample	Microbial diversity	References
1	Primary Schools; Malta	Settled dust was collected using a vacuum, airborne dust collected using electrostatic dust fall collectors, and the floor dust was collected using ALK adaptors and filter cassettes fitted to vacuum cleaners	Bacteria: *Mycobacterium*, *Streptomyces* Fungi: *Alternaria alternata*, *Aspergillus versicolor*, *Cladosporium herbarum*, *Trichoderma viride*, and a larger fungal group comprising *Penicillium*/*Aspergillus*/*Paecilomyces* spp.	(137)
2	Healthcare facilities; Liguria, Veneto, Tuscany, Campania, Lazio, Apulia, Sardinia, and Sicily; Italy	Air sample by active sampling using Surface Air System and Coriolis®μ, and passive sampling by settle plate method	Selective detection of *Legionella* spp. including *L. pneumophila*	(138)
3	Wolatia Sodo University Teaching and Referral hospital; Ethiopia	Air sample by active sampling using a six-stage Anderson cascade impactor	Selective isolation of multi-drug resistant *Acinetobacter baumanii* and *Pseudomonas aeruginosa*	(139)
4	Hospital; Islamabad, Pakistan	Air samples using personal air samplers	Bacteria: *Aerococcus viridans, Bacillus cereus*, *B. subtilis*, *Kocuria kristinae*, *K. rhizophila*, *K. rosia*, *Kytococcus sedantarius*, *Micrococcus luteus*, *M. terreus*, *Pseudomonas stutzeri, Staphylococcus aureus*, *S. cohnii* *S. haemolyticus* Fungi: *Alternaria alternata, Aspergillus flavus*, *A. fumigatus, A. niger*, *Cladosporium*, *Geotrichum*, *Penicillium*, *Ulocladium chartarum*	(140)
5	Office Building; Gliwice, Poland	Air sample using a six-stage Anderson cascade impactor	Bacteria: *Bacillus cereus*, *Enterococcus faecium*, *Gemella haemolysans, Janibacter anophelis/hoylei*, *Macrococcus brunensis, M. equipercicus*, *Micrococcus luteus*, *Staphylococcus xylosus*	(141)
6	Duke University hospital; Durham, North Carolina, United States	Air sample using NIOSH BC 251 personal aerosol sampler	Viruses: influenza A, influenza D, and adenovirus	(142)
7	Glasshouses in a botanical garden; Jagiellonian University, Kraków, Poland	Air sample using a six-stage Anderson cascade impactor	Bacteria: *Arthrobacter*, *Bacillus*, *Curtobacterium*, *Exiguobacterium*, *Kocuria*, *Microbacterium*, *Micrococcus*, *Pseudomonas*, *Solibacillus* Fungi: *Alternaria*, *Aspergillus*, *Cladosporium*, *Fusarium*, *Penicillium*, *Rhizomucor*, *Rhodotorula*, *Scopulariopsis*, *Trichoderma*	(143)
8	University hospitals, super tertiary care hospitals, regional/general (tertiary care) hospitals, and national infectious disease institute; Central Thailand	Air sample using a liquid impinger system	Selective detection of *Mycobacterium tuberculosis*	(144)
9	Historical museum; Egypt	Active air sampling using volumetric Andersen 2-stage impactor and gravimetric air sample by settle plate method; dry deposited dust collected using a dust collector	Fungi: *Alternaria, Aspergillus fumigatus*, *A. niger*, *A. terreus*, *A. versicolor*, *Aureobasidium*, *Chaetomium*, *Cladosporium*, *Eurotium*, *Penicillium*, *Rhizopus*, *Stachybotarys*, *Ulocladium*	(145)
10	Public schools; Helsinki, and Vantaa, Finland	Filter and settled dust sample, Air sample using RCS® High flow touch microbial air sampler, and endotoxin samples from floors using a vacuum cleaner	Fungi: *Aspergillus westerdijkiae*, *A. versicolor, A. flavus, Chaetomium globosum, Dichotomophilus*, *Fusarium*, *Penicillium chrysogenum*, *P. commune*, *P. expansum*, *Rhizopus oryzae, Trichoderma atroviride*, *T. citrinoviride*, *T. longibrachiatum*, *T. trixiae*	(146)
11	Healthcare and care facilities; Nancy, and Rennes, France	Air sample using cyclonic liquid air sampler, Coriolis® μ	Bacteria: *Bacillus cereus, B. licheniformis*, *Kocuria*, *Micrococcus, Pantoea*, *Pseudomonas*, *Staphylococcus hominis*, *S. epidermidis*, *S. saprophyticus*, *S. chromogenes*, *Stenotrophomonas maltophilia* Fungi: *Alternaria*, *Aspergillus*, *Cladosporium*, *Eurotium*, *Penicillium*, *Rhodotorula*	(147)
12	Homes; Cincinnati, Ohio, United States	Floor dust samples using vacuum cleaners	Bacteria: *Acinetobacter lwoffii*, *Alkanindiges illinoisensis*, *Coprococcus eutactus*, *Corynebacterium matruchotii*, *Dialister invisus*, *Lactococcus*, *Massilia*, *Pseudomonas*, *Staphylococcus aureus*, *Streptococcus* Fungi: *Acrimonium fusidioides, A. illinoisensis*, *Candida parapsilosis*, *C. tropicalis, Epicoccum nigrum*, *Toxicladosporium irritans*, *Plectosphaerella oratosquillae*, *Phaeosphaeria podocarpi*, *Rhodotorula mucilaginosa*	(148)
13	Three major hospitals; Kuwait	Aerosol sample using a specialized sampler	Bacteria: *Haemophilus influenzae*, *Mycoplasma pneumoniae*, *Streptococcus pneumoniae* Viruses: SARS CoV-2 virus, non-SARS-coronavirus strains HKU1 and NL-63, respiratory syncytial virus, human bocavirus, human rhinoviruses, human enteroviruses, influenza B virus	(149)
14	Detached house, townhouse, and apartment; Greater Copenhagen, Denmark	Inhalable air fraction collected using Gesamtstaubprobenahme (GSP) conical inhalable samplers, and PM_1_ fraction collected using Triplex cyclones.	Fungi: *Acremonium strictum*, *Aspergillus. fumigatus*, *A. glaucus*, *A. niger*, *A. versicolor*, *Chaetomium globosum*, *Cladosporium cladosporioides*, *C. herbarum*, *C. sphaerospermum*, *Penicillium chartarum*, *P. expansum*, *Rhizopus stolonifer*, *Trichoderma viride*, *Ulocladium chartarum, Wallemia sebi*	(150)
15	University buildings; Johannesburg, South Africa	HVAC-filtered dust and floor dust samples collected using sterile swabs	Bacteria: *Alcaligenes*, *Bacillus*, *Bradyrhizobium*, *Corynebacterium*, *Dietzia*, *Mesorhizobium*, *Mycobacterium*, *Paenochrobactrum*, *Pseudomonas*, *Rhodococcus*	(151)
16	Various hospital wards; Ardabil, Iran	Air sample using an impinger	Specific detection of SARS CoV-2 virus	(152)
17	Gyms; Ardabadil city, Iran	Air sample using the Anderson single-step sampler	Bacteria: *E. coli*, *Pseudomonas*, *Staphylococcus*	(153)
18	Maputo central hospital and microbiology laboratory of the University of Eduardo Mondlane faculty of medicine; Mozambique	Air sample using passive method and surface swab samples	Bacteria: *Acinetobacter baumanii*, *Bacillus* sp., *Burkholderia cepacia*, *Citrobacter koseri*, *C. braakii*, Coagulase-negative staphylococci, *Cronobacter*, *Enterobacter amnigenus*, *E. cloaceae*, *Haemophilus parainfluenzae*, *Klebsiella pneumoniae*, *K. oxytoca*, *Moellerella wisconsensis*, *Moraxella lacunata*, *Pantoea* sp., *Pseudomonas luteola*, *Raoultella ornithinolytica*, *Serratia odorifera*, *S. ficaria*, *S. plymuthica*, *S. rubidaea* Fungi: *Aspergillus flavus*, *A. niger*, *Fusarium verticillioides*, *Mucor* sp., *Paecilomyces variotii*, *Rhizopus*	(154)
19	Shopping Malls; Xiamen, China	Floor and escalator surface swab samples	Bacteria*: Acinetobacter baumanii*, *Enterobacter asburiae*, *E. cloacae*, *Erwinia*, *Leclercia adecarboxylata*, *Pantoea dispersa*, *Staphylococcus aureus* Fungi: Seasonal variations observed in fungal community composition with Eurotiomycetes, Saccharomycetes, and Wallemiomycetes dominating the summer samples and Tremellomycetes, Agaricomycetes, Microbotryomycetes, and Dothidiomycetes dominating the spring samples	(155)
20	Subway stations; Busan, South Korea; Boston, United States; Mexico City, Mexico; Moscow, Russia	Surface swab samples	Bacteria: *Brevundimonas*, *Corynebacterium*, *Cutibacterium*, *Deinococcus*, *Dietzia*, *Janibacter*, *Kocuria*, *Lawsonella*, *Leuconostoc*, *Methylobacterium*, *Micrococcus*, *Rothia*, *Streptococcus*, *Sphingomonas*, *Staphylococcus*, *Stenotrophomonas*	(156)

### 6.1 Bacteria

The most common bacterial genera observed in dust and air samples collected from various types of indoor environments are *Acinetobacter*, *Actinobacteria*, *Arthrobacter*, *Alcaligenes*, *Bacillus*, *Corynebacterium*, *Kocuria*, *Micrococcus*, *Propionibacterium*, *Staphylococcus*, and *Streptococcus* species (43, 45, 140, 147). Barberan et al. (35) indicated that there could be differences in bacterial types isolated depending on the gender occupancy of a particular environment. Relatively, a greater abundance of *Corynebacterium*, *Dermabacter*, and *Roseburia* spp. was found in homes dominated by males, whereas those occupied mainly by females had more *Lactobacillus* spp. Further, seasonal variations are observed in bacterial populations in the indoor environment. In a study by Rintala et al. (157), the relative abundance of alpha- and beta-proteobacteria increased slightly toward summer in house dust samples. The proportion of firmicutes and gamma-proteobacteria was highest in winter and that of actinobacteria, alpha- and beta-proteobacteria in spring or summer, whereas the diversity of *Bacteroides* peaked in fall. Barberan et al. (35) reported that species of *Bacteroides, Porphyromonas*, *Arthrobacter*, *Moraxella*, *Blautia*, and *Neisseria* were associated with dogs, while species of *Prevotella*, *Porphyromonas*, *Jeotgalicoccus*, *Sporosarcina*, *Moraxella*, and *Bifidobacterium* were associated with cats, in household dust samples.

The indoor air of hospitals and healthcare settings can also be a source of harmful bacteria like *Mycobacterium tuberculosis*, *Staphylococcus aureus*, *Escherichia coli*, *Legionella pneumophila*, and *Pseudomonas aeruginosa.* Consequently, patients, healthcare workers, visitors, and mainly immunocompromised patients are at a higher risk of acquiring nosocomial infections (138, 144, 158). In particular, *Legionella pneumophila,* is a highly concerning indoor pathogen, that can easily get aerosolised from toilet or sink faucets, shower heads, bathtubs, plumbing systems, and is abundantly found in various healthcare facilities (159, 160). It has also been found to contaminate the dust in air conditioning and HVAC systems. Both water and air sampling are performed to evaluate the pathogen exposure and to suggest measures for its control. In a study using real time TaqMan PCR, *Legionella pneumophila* was found in 66.7% of the dust samples collected from the air ducts of central air conditioning system (161). Montagna et al. (138) conducted a multicentre study in Italy, to detect airborne contamination of *Legionella* from the water sources. They found that the liquid impingement technique (using Coriolis®μ) was effective in the collection of airborne pathogens combined with molecular investigations in comparison to passive and active dust sampling. Transmission of airborne antibiotic-resistant bacteria like methicillin-resistant *Staphylococcus aureus*, beta-lactam-resistant *Acinetobacter* sp., vancomycin-resistant streptococci, and their inhalable antibiotic-resistance genes in the hospital environment are also a significant threat to public health (162). In a study assessing bacterial contamination of surfaces, medical equipment, and indoor air of the pediatric ward and neonatal intensive care unit of an Ethiopian hospital, a quarter of the bacteria isolated were found to be multi-drug resistant, and most were associated with nosocomial infections (163).

### 6.2 Fungi

Exposure to fungi, their spores, hyphal fragments, and compounds like mycotoxins and beta-glucans in the indoor environment, is considered a major health risk mainly associated with hypersensitivity and allergic risks to the occupants (164). Outdoor air fungi mostly dominate the patterning of indoor air and can easily get suspended in the dust or settle on surfaces of any built environment (22, 32). The indoor temperature range of 10-35°C is highly conducive to the growth of fungi. Fungi can obtain nutrients from plant or animal matter in house dust, building, construction, and painting materials, cooking oil deposits, clothes, books, and paper (47). Dampness is a significant contributor to fungal growth and their spores, fragments, and allergens can easily get aerosolized in the indoor environment (47, 165). Both natural and mechanical ventilations are also considered as likely sources of fungal contamination. High humidity due to condensation of water and settled dust inside air-conditioning ducts, filters, and collecting trays are considered potential sites for high fungal contamination (165, 166). The prevalence of fungi in the indoor air of healthcare facilities poses a high risk of invasive fungal infections. This has been extensively studied and reviewed by Belizario et al. (167). The fungal genera predominating various indoor environments are *Aspergillus*, *Penicillium*, *Fusarium*, *Alternaria*, *Cladosporium*, *Stachybotrys*, *Trichoderma*, and yeasts like *Candida* spp. (137, 154, 168). They have frequently been related to hospital-acquired infections and exacerbation of respiratory symptoms (20, 167). The microbial content of rug dust and vacuum cleaner bag, dust samples determined by qPCR showed *Penicillium*, *Aspergillus*, and *Paecilomyces* to be present in the highest concentrations (169).

### 6.3 Viruses

Though bacteria and fungi are monitored frequently in various indoor environments, not many efforts have been made to investigate the viral diversity of the indoor air (101, 133). Respiratory infections, like the common cold, bronchiolitis, and influenza, can transmit through aerosolized droplets (170, 171). Respiratory syncytial, influenza, parainfluenza, corona, and adeno viruses are implicated in these infections (172). Low relative humidity and cold temperatures are believed to increase the transmission of respiratory viruses (173, 174). Human activities like breathing, coughing, sneezing, talking, and laughing release viral aerosols abundantly in the indoor air (175). Moon et al. (176) showed that the indoor air of residential apartments in South Korea mainly contained adenoviruses and influenza A virus, with concentrations of adenoviruses higher in winter than in spring. In another study by Yang et al. (177), one-third of the samples from the health center and two-thirds from the day-care center were confirmed to contain aerosolized influenza A virus.

The diversity of viruses in the indoor environment could be beyond human viruses. Rosario et al. (133) used a metagenomic approach to investigate the diversity of DNA and RNA viruses in the dust samples accumulated in HVAC filters of university dormitory rooms. They detected a broad diversity of viruses, including human papillomaviruses (HPVs) and polyomaviruses (HPyVs), which were found to be associated with a range of hosts. Rare zoonotic diseases caused by deadly viruses like Marburg, Ebola, Hanta, and Lassa can pose a potential nosocomial threat (178). During the SARS outbreak of 2003, infectious viral droplets entered buildings via sewage and drainage systems due to inadequacies in plumbing (179). The profuse transmission of SARS-CoV-2 and cluster cases were primarily due to the confined, crowded, and poorly ventilated indoor environment (180).

## 7 Microbe-associated health risks in the indoor environment

Exposure to microbes in the indoor environment can lead to adverse health effects like respiratory illnesses, including allergies, microbial infections, acute toxic effects, and cancer, as described below. The major routes of human exposure to airborne microorganisms and their products are mainly inhalation followed by ingestion and dermal contact (15). Various health effects following microbial exposure depend on the dose, route, and timing of exposure and the person’s genetic disposition (181). Molds and bacteria can also produce components like endotoxin, beta-1-3-glucan, muramic acid, ergosterol, allergenic proteins, volatile organic compounds of microbial origin (MVOCs), and mycotoxins (32, 182, 183) which can also pose additional health hazards.

### 7.1 Respiratory illnesses, allergies, and hypersensitive reactions

Exposure of the respiratory tract to microbes or their products can lead to mucous membrane irritation, allergic rhinitis, asthma, bronchitis, organic dust toxic syndrome (ODTS), chronic obstructive pulmonary disease (COPD), or allergic alveolitis (184, 185). Moldy and dusty environments are highly uncomfortable to susceptible individuals, with fungi such as *Aspergillus*, *Penicillium*, *Cladosporium, Acremonium*, *Paecilomyces,* and *Mucor* found to be commonly associated with respiratory infections and allergies (168). Asthma is common in children, with allergens and molds influencing the development of the disease (137). Fungal DNA has also been considered a risk factor for childhood asthma at home (181). However, according to hygiene hypothesis, exposure to microbes in early childhood could protect against allergies and asthma by stimulating the immune system, and that excessive cleanliness in the human environment might not necessarily be beneficial (186). Among fungi, *Aspergillus* species are the most commonly found indoor fungi. *Aspergillus fumigatus* and *Aspergillus flavus* are frequently encountered and responsible for sinusitis, allergic broncho-pulmonary aspergillosis (ABPA), and hospital-acquired infections (187, 188). High fungal or dust exposure has been linked to hypersensitive pneumonitis (189). Fungal metabolism produces volatile compounds such as 3-methyl furan that can irritate eyes, nose, and airways leading to headache, nausea, dizziness, and fatigue (40, 190). Inhalation of high doses of endotoxins, glucans, fungal spores, and mycotoxins can contribute to airway irritation and inflammation, decrease lung function, exacerbate asthma, and chronic conditions like ODTS and COPD (164, 191).

### 7.2 Infections

Airborne transmission is the most likely route for many microbial infections in the indoor environment. *Legionella* bacteria causing Pontiac fever and highly fatal pneumonia can rapidly spread in indoor environments through aerosolized contaminated water and inhalation of infected mist or vapor (138). *Mycobacterium tuberculosis* can be transmitted by inhaling the infectious droplet nuclei expectorated from sputum-positive patients in the indoor air of hospitals or other environments (192). *Staphylococcus aureus*, *Pseudomonas aeruginosa*, *Enterococcus faecalis*, *Enterococcus faecium*, *Acinetobacter baumanii*, and *Escherichia coli* are some of the most frequently encountered bacteria in the indoor air of hospitals, causing various nosocomial infections (193).

Immunocompromised patients are at potential risk for more severe opportunistic and systemic fungal infections caused by *Blastomyces*, *Coccidioides*, *Cryptococcus, Histoplasma, Alternaria*, *Heliminthosporium*, *Cladosporium*, *Fusarium, Aspergillus*, *Phoma*, and *Penicillium* (194, 195). Similarly, the presence of these fungi in the indoor air of critical hospital areas, especially neonatal and pediatric ICU, has been linked to the increased incidence of mucocutaneous colonization and a high risk of invasive fungal infections in neonates (167).

Human corona, respiratory syncytial, rhino, adeno, measles, mumps, rubella, and enteric viruses are readily transmitted through air droplets in the indoor environment causing various infections (196–198). The COVID-19 pandemic has recently shown the extreme contagiousness of the Severe Acute Respiratory Syndrome Coronavirus 2 (SARS-CoV-2) virus in the indoor environment, which has been responsible for high morbidity and mortality (199). A special section is dedicated here to the potential implications of poor indoor air quality in transmitting SARS-CoV2.

### 7.3 Cancer

Several studies have tried to ascertain the link between cancer and bioaerosol exposure (200–202). Mycotoxins have been established as non-viral biological carcinogens (203, 204). Among them, aflatoxin from *Aspergillus flavus* is the best-known carcinogenic mycotoxin, particularly linked to liver cancer (205, 206). Ochratoxin A is also a possible human carcinogen (183, 207). The lung cancer risk of workers in the meat/poultry industry was reviewed with evidence pointing to oncogenic viruses associated with animals and heavy exposure to airborne material, including micro-organisms, fecal material, dander, and feathers (201).

## 8 Indoor air pollution from the COVID-19 perspective

The global pandemic of COVID-19 caused by the SARS-CoV-2 has left an unprecedented impact on various facets of human life, including health, lifestyle, economy, and the environment (208, 209). Terms like disinfection, masks, social distancing, and quarantine have become a part of our daily life. On the one hand, a few studies indicated improvement in ambient air quality in many cities during the lockdown, attributed to reduced industrial activities, vehicular movements, and various human activities (210–213). On the other hand, the reduced outdoor pollution did not contribute to any health gains, as prolonged exposure to polluted indoor air negated this effect (214, 215). Poor indoor air quality increased COVID-19 infection and viral transmission in enclosed spaces (216). It took several months before the WHO acknowledged the airborne transmission of SARS CoV2, especially when people spent prolonged periods in indoor, crowded, inadequately ventilated spaces (217). The aerosolized virus is stable for 3 h and can travel long distances in closed and open environments (214). These aerosols need to be controlled to reduce the risk of new infections. Also, in a study conducted in Bergamo, Northern Italy, viral RNA was isolated from the particulate matter, possibly explaining a higher COVID-19 burden in areas with high air pollution (218).

People were compelled to stay inside owing to home quarantine and lockdowns. This may have impacted indoor air quality due to insufficient ventilation, overcrowding, recirculation of polluted air, and increased household activities and office work (214, 215). Further, aerosolized virus released from infected people quarantined at homes, or hospitals could have increased the risk and deteriorated the indoor air (219). A study showed that the cough from an individual with a high SARS-CoV-2 load could contain 7.44 million viral copies/m^3^ (220). If the quality of the air is poor to begin with, people may be inadvertently exposed to various SBS-related symptoms (221) putting people with pre-existing medical conditions at a higher risk. Due to the high transmission of the SARS-CoV-2 virus in indoor spaces, IAP has thus become a table talk worldwide (180). The COVID-19 pandemic has thus strongly taught us to focus equally on sustainable and safe indoor spaces, as poor indoor air, can likely intensify the impact of air-borne viral outbreaks and other microbial infections (222, 223).

## 9 Indoor air pollution in India

In developing countries, including India, people are exposed to the highest air pollution in the indoor environment (224, 225). There is a general lack of awareness among people in India as air pollution is only thought to be associated with the outdoor environment, and the inhabitants are considered safe indoors (18). Here, rural and urban poor populations still use simple solid fuels that release abundant amounts of suspended particulate matter, harmful chemicals and gasses and have been associated with severe respiratory and other health risks (226). Overcrowded and confined indoor spaces, improper ventilation, inadequacies in building design, and various socio-economic factors also aggravate poor indoor air quality (213, 227). Microbial contaminants also significantly contribute to IAP; however, in India, this aspect has not been dealt with extensively (228) and can pose various health hazards to the occupants of the indoor environment, as discussed earlier.

Further, there are no standard guidelines to monitor and assess various parameters related to IAP, including microbiological assessment (18). The Central Pollution Control Board (229) under the Ministry of Environment and Forests (MoEF), Government of India, does not include IAP in its agenda and mainly focuses on outdoor air pollution. Thus, this knowledge and skill gap is highly concerning (18). Damp, overcrowded houses, and poorly ventilated buildings are a hot spot for microbial contaminants and a source of respiratory illnesses like tuberculosis, contributing significantly to the national disease burden (230). India hosts diverse climatic conditions, and thus the indoor microflora diversity and concentration show a lot of seasonal variations (5, 231, 232). Some representative studies on the isolation and assessment of microflora in various indoor environments conducted in India’s urban and rural settings are highlighted in Table 2.

**Table 2:** Studies on microbiological assessment of the indoor environment in India (2012-2022).

S. No.	City/State/Rural or Urban	Type of Indoor environment	Purpose of the study	Sampling method	Culture media	Microbial diversity	Reference
1	Not specified	Maxillofacial operation theater of a teaching dental hospital in India	Assessment of contamination of indoor air and surfaces	Active sampling by centrifugal air sampler method; Passive sampling by settle plate method; Surface Swabbing	NA and BA for bacteria; PDA for fungi	Bacteria.: *E. coli, Proteus, S. aureus, Streptococcus* beta*-hemolyticus* Fungi: *Aspergillus*, *Fusarium*	(233)
2	West Chennai/Tamil Nadu/Urban	Orthopedic Ward of a tertiary healthcare facility	Characterization of indoor bioaerosols in a hospital	Passive sampling by exposed-plate gravitational method; Active sampling by filtration (personal sampler with gelatin filters) and impingement (BioSampler)	5% Sheep BA and MA for bacteria; SDA for fungi	Bacteria: Coagulase-negative staphylococci, diphtheroids, micrococci, *Enterobacte*r, and *Pseudomonas* Fungi: *Aspergillus flavus*, *A. fumigatus*, *A. niger*, *A. terreu*s Absidia, *Candida krusei*	(76)
3	Jodhpur/Rajasthan/Urban	Senior secondary school	Quantitative and qualitative determination of airborne microorganisms and to study their seasonal variability	Passive sampling by plate exposure method	NA for bacteria; PDA supplemented with chloramphenicol for fungi	Bacteria: *Bacillus lentus*, *Bacillus megaterium*, *Bacillus subtilis*, *Enterobacter aerogenes*, *Escherichia coli*, *Micrococcus kristinae*, *Micrococcus luteus*, *Pseudomonas*, *Serratia marcescens*, *Staphylococcus aureus*, *Staphylococcus epidermidis*, Fungi: *Alternaria*, *Aspergillus flavus*, *A. fumigatus*, *A. niger*, *A. terreus*, *Cladosporium*, *Fusarium*, *Helminthosporium*, *Rhizopus*	(234)
4	Delhi/India/Urban	Various areas of Jawaharlal Nehru University Library	Estimation and identification of Bioaerosols	Active sampling by BUCK Bio-culture pump	EMB and BA for bacteria; PDA for fungi	Bacteria: *Bacillus*, *Micrococcus*, *Streptococcus* Fungi: *Aspergillus flavus*, *A. nidulans*, *Cladosporium*, *Curvularia*, *Penicillium*, *Rhizopus oryzae*	(235)
5	Delhi/India/Urban	School of Environmental Sciences building, Jawaharlal Nehru University	Determination of bacterial concentrations and to assess their correlation with meteorological parameters	Active sampling using a handy air sampler	LB broth and agar with cycloheximide	Gram-positive rods, Gram-positive cocci, Gram-negative rods, Gram-negative cocci (Bacterial genera not identified; only bacterial counts as CFU/m^3^ determined)	(236)
6	Visakhapatnam/Andhra Pradesh/Urban	Primary and secondary schools (classrooms, office rooms, libraries, canteens, and toilets)	Assessment of microbiological indoor air quality	Passive sampling by settle plate method	EMB for bacteria; PDA for fungi	Bacteria: *Bacillus*, *Escherichia coli*, *Micrococcus*, *Pseudomonas*, *Serratia*, *Staphylococcus aureus* Fungi: *Aspergillus flavus*, *Mucor*, *Rhizopus*, *Alternaria*, *Penicillium*, *Cladosporium*	(237)
7	Kalyani/West Bengal/Urban	Hospital (general ward, female ward, children’s ward, and operation theater)	Environmental monitoring of hospital aero microflora	Passive sampling by settle plate method	NA for bacteria; PDA for fungi	Bacteria: *Escherichia coli*, *Klebsiella*, *Pseudomonas aeruginosa*, *Staphylococcus aureus* Fungi: *Aspergillus*, *Candida*, *Fusarium*, *Penicillium*	(238)
8	Manipal/Karnataka/Urban	ICUs in a tertiary care hospital	Assessment of microorganisms and their antimicrobial susceptibility patterns in microbial in relation to the nosocomial infections	Passive sampling by Settle plate method	Blood agar medium	Bacteria: *Acinetobacter*, *E. coli*, *Klebsiella*, *Micrococcus*, *Pseudomonas*, *Staphylococcus* Fungi: *Aspergillus*	(239)
9	Nagpur/Maharashtra/Urban	House	Investigation of fungal flora	Passive sampling by plate exposure method	PDA and Peptone dextrose agar	Fungi: *Aspergillus alternata*, *A. flavus*, *A. fumigatus*, *A. niger*, *A nidulans*, *Fusarium oxysporum*, *Penicillium chrysogenum*	(240)
10	Triplicane, Chennai/Tamil Nadu/Urban	Public toilet	Investigating the presence of airborne fungi in public toilets	Exposed Plate Technique	SDA for fungi	Fungi: *Aspergillus flavus*, *A. fumigatus*, *A. niger*, *A. terreus*, *Rhizopus oryzae*	(241)
11	Jodhpur/Rajasthan/Urban	Outdoor patient room of a three-story private hospital	Evaluation of the variability and the effect of meteorological parameters on airborne fungi	Passive sampling by settle plate method	PDA for fungi	Fungi: *Aspergillus flavus*, *A. fumigatus*, *A. niger*, *A. solani*, *Cladosporium herbarum*, *Fusarium oxysporum*, *Helminthosporium* sp., *Rhizopus*	(242)
12.	Dehradun/Uttarakhand/Urban	Doon hospital and combined medical institute	Investigating the airborne microbial population	Passive sampling by exposed plate technique	NA for bacteria; PDA for fungi	Bacteria: *Bacillus*, *E. coli*, *Micrococcus*, *Staphylococcus* Fungi: *Alternaria*, *Aspergillus*, *Cladosporium*, *Penicillium*, *Rhizopus*	(243)
13	Moga/Punjab/Urban	Private maternity home	Assessment of microbial contamination	Passive sampling by settle plate method	NA, Cetrimide Agar, Hicrome™ *Bacillus* agar, MA, MSA, Sheep BA, Anaerobic BA for bacteria; PDA for fungi	Bacteria: *Bacillus*, *Enterobacteriaceae*, *Pseudomonas*, *Staphylococcus* Fungi: *Absidia*, *Aspergillus*, *Exophiala*, *Mucor*, *Penicillium*, *Rhizopus*	(244)
14	Pune/Maharashtra/Urban	Hospital (pediatric ward, maternity ward, labor room, pediatric intensive care unit and neonatal intensive care unit)	Assessment of bacterial contamination and their relationship with nosocomial infections	Passive sampling by settle plate method	MA for bacteria	Bacteria: *Escherichia coli*, *Pseudomonas aeruginosa*, *Staphylococcus aureus*	(245)
15	Chennai/Tamil Nadu/Urban	Diverse indoor environments including laboratory, student office, air-conditioned room, eatery, and residential apartment	Assessment of bioaerosols	Active Sampling by six stage anderson sampler	TSA for bacteria; PDA for fungi	Bacteria: *Actinobacteria*, Alphaproteo-bacteria, Betaproteo-bacteria, Gammaproteo-bacteria, Dinococci, Bacillii, Flavobacteria Fungi: *Bipolaris*, *Cladosporium*, *Aspergillus*, *Alternaria*, *Cochliocolus*, *Curvularia*, *Drechslera*, *Fusarium*, *Mucoromycotina*, *Nigrospora*, *Penicillium*, *Purpuriocillium*, *Rhizopus*, *Trichosporon*	(246)
16	Jammu/Jammu and Kashmir/Urban	Banks located in different areas	Evaluation of bacteria and fungi in relation to suspended particulate matter and relative humidity	Active sampling using a handy air sampler	NA, MA and BTB Lactose Agar for bacteria; PDA and Czapek Dox Agar for fungi	Bacteria: *Acinetobacter*, *Bacillus*, *E. coli*, *Klebsiella*, *Micrococcus*, *Pseudomonas*, *Staphylococcus aureus* Fungi: *Alternaria*, *Aspergillus fumigatus*, *A. glaucus*, *A. niger*, *A. versicolor*, *Bipolaris*, *Cladosporium*, *Curvularia*, *Fusarium*, *Mucor*, *Penicillium*, *Rhizopus*, *Saccharomyces*	(247)
17	Delhi/Urban	Slum, residential, and plush urban areas near the University of Delhi	Spatio-temporal variations in bioaerosol concentrations in the indoor environment of different socio-economic zones	Passive sampling	BA for bacteria; PDA for fungi	Only bacterial and fungal counts were determined.	(248)
18	Israna/Haryana/Rural	N.C. Medical College and Hospital (eye, general, ENT, orthopedics, gynecology, and emergency operation theaters)	Studying prevalence and type of bacterial contamination	Passive sampling by settle plate method and surface swabbing	NA, MA, BA for bacteria	Bacteria: *Bacillus*, *Coagulase-negative* Staphylococci, *Escherichia coli*, *Klebsiella*, Micrococci, *Pseudomonas*, *Staphylococcus aureus*, *Streptococcus* Fungi: *Aspergillus*, *Penicillium*	(249)
19	Puducherry/Urban	Tertiary care hospital (general medicine, general surgery, obstetrics, gynecology, and orthopedics departments)	Assessment of prevalence rate of various microorganisms	Passive sampling by settle plate method	Sheep BA for bacteria; SDA for fungi	Bacteria: *Bacillus*, *Diptheroides*, *Micrococcus*, *S. aureus* Fungi: *Aspergillus flavus*, *A. fumigatus*, *A. niger*, *Fusarium*	(250)
20	Delhi/Urban	Dr. B.R. Ambedkar central library and Central laboratory animal resources, Jawaharlal Nehru University	Assessment of prevalence and antibiogram of *Staphylococcus*	Passive sampling by settle plate method; Active sampling using biosampler	EMB for Gram-negative bacteria, Sheep BA for Gram-positive bacteria and MSA for staphylococci	Bacteria: *S. aureus*, *S. capitis*, *S. cohnii*, *S. epidermidis*, *S. haemolyticus*, *S. hominis*, *S. lentus*, *S. saprophyticus*, *S. sciuri*, *S. warneri*, *S. xylosus*	(251)
21	Jalna/Maharashtra/Urban	Indian Institute of Medical Sciences and Research (surgery, emergency, orthopedic, general, obstetric, medical, and tuberculosis wards)	Assessment of bioaerosols	Passive sampling by settle plate method	NA and blood agar for bacteria; SDA for fungi	Bacteria: *Bacillus*, *Clostridium*, *Coccobacilli*, *Diphtheroids*, *E. coli*, *Klebsiella*, *Micrococci*, *Pseudomonas*, *Staphylococcus aureus* Fungi: *Aspergillus*, *Candida*	(64)
22	Kyasaram/Telangana/Rural	Living rooms of rural houses	Assessment of ambient concentrations of airborne microbes and endotoxins	Active Sampling by single-stage cascade impactor	TSA for bacteria; PDA for fungi	Bacteria: *Bacillus*, *B. anthracis*, *Enterobacter cloacae*, *Micrococcus*, *Staphylococcus*, Fungi: *Alternaria*, *Aspergillus*, *Penicillium*	(252)
23	Kolkata/West Bengal/Urban	Outdoor unit, newborn baby ward, respiratory care unit, step downward, and thalassemia care unit of government children hospital	Assessment of airborne fungi	Passive sampling by petri dish gravitational method	MEA for fungi	Fungi: *Alternaria*, *Aspergillus flavus*, *A. fumigatus*, *A. niger*, *A. sydowii*, *Cladosporium herbarum*, *Cladosporium*, *Corynespora cassicola*, *Curvularia lunata*, *Curvularia pallescens*, *Fusarium*, *Humicola grisea*, *Mucor*, *Penicillum*	(231)
24	Delhi/Semi-urban/Urban	Residential areas (slum area), Wazirpur industrial area, and other commercial areas	Determination of microflora composition, diversity, and size distribution with regard to seasonal variation	Active sampling by Anderson six stage sampler	TSA with cycloheximide for bacteria; SDA with rose bengal dye for fungi and	Bacteria: *E. coli*, *Klebsiella*, *Micrococcus*, *Pseudomonas*, *Spirillum*, *Staphylococcus*, *Streptobacillus*, *Streptococcus* Fungi: *Alternaria*, *Aspergillus flavipes*, *A. flavus*, *A. fumigatus*, *Candida*, *Cladosporium*, *Fusarium*, *Microsporum*, *Mucor*, *Penicillium*, *Rhizopus*, *Saccharomyces*, *Trichoderma*	(232)
25	Delhi/Urban	Residences, college classrooms, coaching academies, godowns, Research Laboratories	To investigate health risks among the people living in industrial, educational, and residential areas due to poor microbial indoor air quality	Passive sampling by settle plate method	TSA with cycloheximide for bacteria; SDA with chloramphenicol for fungi	Bacteria: *E. coli*, *Micrococcus*, *Pseudomonas*, *Staphylococcus*, *Streptobacillus*, *Streptococcus* Fungi: *Alternaria*, *Aspergillus*, *Candida*, *Cladosporium*, *Fusarium*, *Mucor*, *Penicillium*, *Rhizopus*	(253)

BA: blood agar, BTB Lactose agar: bromo thymol blue lactose agar, EMB: eosin methylene blue agar, LB: Luria-Bertani, MA: MacConkey agar, MEA: malt extract agar, NA: nutrient agar, PDA: potato dextrose agar, SDA: Sabouraud’s dextrose agar, TSA: trypticase soy agar.

However, it is clear from these studies that primarily passive sampling procedures combined with conventional culturing methods have been used. Thus, a large proportion of non-culturable microbes still remain unassessed. There is a need to introduce advanced molecular and spectrometric techniques to routinely monitor microbiological indoor air quality and bridge this knowledge gap. Further, mainly bacterial and fungal contaminants have been studied, with only a few studies assessing viruses and microbial products such as endotoxins, mycotoxins, and microbial allergens in the indoor environment (252, 254).

## 10 Strategies for microbial control in the indoor environment

Various mitigation strategies aim to control microorganisms and improve indoor air quality by optimizing ventilation systems, controlling emission sources, and developing air purification technologies, as discussed here (255).

### 10.1 Ventilation

Ventilation can help remove or dilute pollutants and control humidity or dampness in the built environment, thereby maintaining the health and comfort of the occupants (256, 257). International agencies such as ASHRAE (American Society of Heating, Refrigerating, and Air-Conditioning Engineers), WHO, REHVA (Representatives of European Heating and Ventilation Associations), and CDC (Center for Disease Control and Prevention) have also recognized that the transmission of airborne diseases can be effectively managed by ventilation (257–259). For instance, CDC (258) has provided detailed guidelines including cost considerations for improving ventilation in buildings. Ventilation can be provided by natural or mechanical means (260, 261). Natural or passive ventilation is achieved through airflow provided by doors, windows, louvers, and vents (7, 259). Various studies have reported that natural ventilation is effective in controlling the transmission of the infectious airborne microbes (260, 262). In low resource healthcare settings, Escombe et al. (263) demonstrated that it could be used as a low-cost and energy efficient measure to reduce the risk of transmission of nosocomial tuberculosis. Further, the rampant spread of corona virus brought natural ventilation strategy to the forefront of the discussion as a crucial factor that can help in lowering the viral build-up in the indoor spaces. In this context, Vignolo et al. (264) qualitatively assessed airborne viral transmission and natural ventilation in school classroom in Uruguay and reported that periodic ventilation can act as a useful strategy to reduce transmission of airborne microbes. However, in another study, no impact was reported (265). Despite the low cost, energy efficiency, low maintenance, and higher airflow changes, natural ventilation is difficult to maintain in a tight building without cross ventilation. Further, it is not a feasible strategy for healthcare facilities and countries with colder climates as natural ventilation can increase the thermal discomfort for the occupants (266). Thus, a cross-sectional study involving all the factors can potentially give an insight for optimal use of natural ventilation.

Mechanical ventilation uses an air handling system like HVAC that circulates fresh and recycled air via ducts (261). Healthcare facilities with efficiently maintained and operated mechanical air conditioning, and ventilation systems were found to be less contaminated than buildings with naturally ventilated systems (267). In a study by Gołofit-Szymczak and Górny (268), different ventilation systems were assessed for their impact on microbiological indoor air quality of 15 office buildings. Higher concentrations of both bacteria and fungi were observed in naturally ventilated office in comparisons with offices having air-conditioning and mechanical ventilation systems. Similarly, HVAC system was found to effectively remove fungal spores (269). However, it is essential to note that mechanical ventilation systems may be a source of biological contamination, as discussed earlier. Further, the high transmission of SARS-CoV-2 in indoor spaces has made us all think critically about indoor air quality, especially in hospitals, and the approaches to controlling nosocomial infections (270). Fonseca et al. (271) suggested that the mechanical ventilation systems in healthcare facilities could be complemented with natural ventilation to adequately control relative humidity and CO_2_ concentrations to minimize the risk of airborne infections. Both mechanical and natural ventilation systems combined in a hybrid technology can also provide sustainability in terms of energy efficiency (272), and the shortcomings of natural ventilation can be overcome by the mechanical components (273). The frequency of regular monitoring, cleaning, and maintenance procedures can be adjusted based on operating hours and human occupancy to achieve good air quality (20).

Studies show that HVAC filtration can effectively control pathogens penetrating the building envelope and their transmission in the indoor environment (274, 275). Thus, to improve the performance of HVAC systems, panel filters are installed in their ducts (274, 276, 277). These filters are installed as per the standard guidelines and used widely (278, 279). HEPA or High-Efficiency Particulate Air filters must be cleaned and replaced periodically to maintain proper function (280). By following the air route and sources of contamination through sequential sampling, Cabo Verde et al. (20) assessed the efficacy of HEPA filters installed in the ventilation systems of operation theaters and found an effective reduction in bacterial concentration from outdoors to indoors. These filters have the limited ability to inactivate the collected biological agents, which may regrow with adequate humidity levels (281). Nanofiber filters can overcome this problem and are very effective in indoor environments where stringent air purification is needed, such as hospitals, food, and pharmaceutical industries (282).

The inactivation of microbial contaminants in indoor air could also be brought about by utilizing other methods integrated with the HVAC system (275, 280, 283). These include ultraviolet germicidal irradiation (UVGI) (284, 285); chlorine dioxide gas (286); antimicrobial compounds (287); electrostatic technology (288); photocatalytic oxidation (289); plasma cluster ion technology (290); microwave heating (291); ozone sterilization (292, 293); and dispersion of atomized nanoparticles in the air (294). UVGI system uses UVC rays (200–280 nm) to form photo dimers in nucleic acids to bring about disinfection and has been widely recognized and recommended for installation, especially in healthcare facilities (295, 296). However, it is pertinent to mention that ozone-based technologies can themselves pose an additional health risk by releasing ozone as a harmful by-product (297). Recently, silver nanoparticles and multiwall carbon nanotubes coated with hybrid polypropylene nano-filter were tested in air-conditioning and found to be very effective in killing *Pseudomonas aeruginosa*, *Salmonella enterica*, and *Staphylococcus aureus* (298). Thus, two or more such technologies can be synergized to improve the efficient functioning of the HVAC systems to remove microbial contaminants (283, 299).

### 10.2 Air cleaners and purifiers

Air Purifiers/Cleaners (AP) have become essential for reducing IAP. There has been a considerable increase in sales of air purifiers from 0.8 million units in 2015 to almost 2 million units in 2018, with a similar projection for the future (300, 301). Portable air purifiers utilizing HEPA filtration are commonly used for homes. Air purifiers’ advantages are simple installation, portability, and the lack of harmful by-products (302). Their configuration generally employs a multilayer filter system consisting of a prefilter, a carbon filter, an antibacterial filter, and a HEPA filter (255, 303). Also, APs with disinfection capability are more effective than those with only HEPA filtration (216). In these, UVGI lights are installed within the body of the air cleaners. Combined HEPA and UVGI, air cleaning technologies could effectively remove airborne pathogens (304, 305). Air purifiers or cleaners based on ionization, electrostatic precipitation, cold plasma generation, and photocatalytic oxidation are emerging technologies (216, 255, 306–309). However, currently, no single technology can sufficiently achieve “cleaner” indoor air. Current research is focused on combined and innovative alternatives, such as plasma-catalytic hybrid systems, hybrid ozonation systems, and biofilter-adsorption systems, that can work synergistically to achieve optimum indoor air quality (310–312).

### 10.3 Housekeeping measures

The frequency of housekeeping can also help in lowering the microbial content of the indoor air. This includes basic measures like dusting with a damp cloth or using electrostatic cloth, vacuuming, carpet and rug cleaning, and removal of shoes while entering the house. Persistent dampness and moldy growth on indoor surfaces and in building structures can be minimized. For instance, USEPA has provided guidelines for the control of moisture and mold in homes (313). Franke et al. (314) carried long-term monitoring of a building for biological, chemical, and particulate matter, following a standardized cleaning program. This improved house-keeping initiative was found to be effective in the reduction of culturable bacteria and fungi, airborne dust mass and total volatile organic compounds. However, barring a few reports, there are no direct studies that have ascertained the link between house-keeping and indoor microbial concentrations. This is definitely a thrust area that will also aim to increase awareness about the impact of poor indoor air quality on an individual’s health especially children and older people and those with underlying health conditions.

## 11 Conclusion

Rapid industrialization and urbanization have led people to spend most of their time indoors in tight-built environments. Thus, indoor air quality plays a significant role in their general state of health and comfort. They are exposed to a variety of pollutants, including microorganisms. A vast ecological niche has been created for these minuscule organisms leading to their presence in various microhabitats of the indoor environment, thus presenting us with a complex ecosystem that requires a more profound understanding. Compared to bacteria and fungi, there are limited studies on the viral diversity of the indoor environment. Protocols for sampling and assessing microorganisms need to be standardized and validated as the threshold limits can vary from one indoor environment to another, region to region, and country to country. Culture-independent methods can complement culture-based methods in comprehensively monitoring microbial composition and community structure. Advancements in metagenomic next-generation sequencing approaches have paved the way for better analysis of viral diversity which has been relatively unexplored in comparison to bacteria and fungi. To minimize the knowledge gap, a repository of microbial sequences obtained from different kinds of built environments must be maintained as a referential database source.

Developed countries mostly follow specific guidelines and standards for building design and management of indoor air quality. However, the situation is grave in developing countries with a high disease burden aggravated by various socio-economic factors. A concerted effort is needed to raise awareness about the issue, as simple source control measures can make a massive difference in the quality of the built environment. For example, basic housekeeping measures to prevent dust build-up or humidity control can curtail microbial growth, for which people need to be educated through awareness programs and surveys. Cross-sectional and evidence-based studies will provide a realistic analysis of the microbial impact and help design feasible mitigation strategies. We need to focus on hybrid air cleaning technologies and sustainable building architecture that combines energy saving with appropriate ventilation to minimize the buildup of indoor air pollutants. A multifactorial interdisciplinary approach will involve a synergy between microbiologists, public health experts, engineers, architects, environmental agencies, and the government to devise sustainable solutions to this serious public health issue.

## Author contributions

HC: Writing – original draft, Data curation, Investigation. PA: Data curation, Writing – original draft, Writing – review & editing. KG: Investigation, Writing – original draft. NB: Data curation, Investigation, Writing – review & editing. SV: Investigation, Writing – review & editing. TB: Writing – original draft. AM: Writing – review & editing. RM: Conceptualization, Supervision, Writing – original draft.
